# Determination of reference intervals for common liver function tests among healthy adults

**DOI:** 10.1038/s41598-025-97545-8

**Published:** 2025-04-15

**Authors:** Abdul Baset Abbas, Ashraf Yahya, Zakaria Aloqab, Ahmed AlHudhaifi, Adhwaa Abdu Alateef, Amaturahman Morshed, Azal Qasem, Mohammed Al-Awlaqi, Shahira Alshahari, Nosiba Mohammed, Kholah Mohammed

**Affiliations:** Medical Laboratories Department, Faculty of Medicine and Health Sciences, Ibb University, Ibb City, 70270 Yemen

**Keywords:** Liver function tests, Reference intervals, Bilirubin, Albumin, Protein, ALT, ALP, AST, Immunology, Microbiology, Biomarkers, Medical research

## Abstract

Reference intervals (RIs) are significant means for health evaluation, prognosis, diagnosis, and monitoring of adverse events. The RIs are affected by ethnicity, age, gender, geographic area, diet, socioeconomic, and physical situation. This study aimed to determine RIs of commonly used liver function tests (LFT) for healthy adults in Ibb City, middle of Yemen. A total of 390 participants aged between 18 and 70 were selected and administered a questionnaire. Blood specimens were assembled after an overnight fast, and the sera were separated for analysis of common LFT (DBIL, TBIL, ALB, TP, ALP, AST, and ALT) using Mindray BS-240 Automatic Clinical Chemistry Analyzer. The data were computed by GraphPad Prism 8.0.1. This study revealed that RIs for males and females of DBIL, TBIL, ALB, ALP, and ALT were significantly higher in males than females. Although RIs for TP and AST were higher in males than females, the difference was non-significance. Notably, most of the RIs in our study were different than those from other countries, either higher or lower. In conclusion, this study has established a panel of locally relevant RIs for commonly used LFT in adults, Ibb City, which may help interpret laboratory results for healthy adults and patients.

## Introduction

Reference intervals (RIs) are numerical ranges previously designed along with diagnostic laboratory results to assist in their interpretation. RIs are commonly referred to as the normal range^1^. RIs are typically drawn from a group of healthy individuals, allowing analytical measurements to be interpreted clinically. By comparing results to these intervals, healthcare professionals can better understand the implications of the measurements for patient health^2^.

RIs represent a selection of results, typically encompassing the middle 95%, and are determined based on healthy-defined reference individuals^2^. The International Federation of Clinical Chemistry and Laboratory Medicine (IFCC) defines RI as a group of “reference individuals” selected according to specific criteria, including laboratory investigations, medical history, or physical examinations^3^. These definitions have been supported by prominent organizations such as the World Health Organization (WHO), the International Council for Standardization in Hematology (ICSH), and the Clinical and Laboratory Standards Institute (CLSI)^1^.

The liver is the body factory which is responsible for metabolite detoxification, digestive enzyme production, protein synthesis, red blood cell (RBC) regulation homeostasis, and glucose synthesis and storage^4,5^. Liver function tests (LFT), including hepatic enzymes such as aspartate aminotransferase (AST) and alanine aminotransferase (ALT) are used to evaluate hepatocyte injury^6,7^. Alkaline phosphatase (ALP) assesses hepatic obstruction and gamma-glutamyltransferase (GGT) is a marker of alcoholic hepatitis or biliary obstruction^6^. Additionally, bilirubin (total, direct, or indirect) is a marker of hepatic excretion capacity and metabolism^8^, whereas total protein and albumin impart information on liver synthetic function^9^. LFT can classify a differential diagnosis of the hepatic injury^10^, monitor the progression of disease and treatment efficacy^6^.

To precisely and accurately LFT, one demands precise RIs from a proper population. The RIs are affected by ethnicity, age, gender, geographic area, diet, socioeconomic, and physical conditions^6,11^.

Furthermore, appropriate RIs are the most critical elements for a health evaluation, therapy monitoring, disease diagnosis, and prognosis assessment^12^. It is important to note that the dissimilarities in RIs across different countries cannot be generalized for the whole population^13^. Furthermore, the International Federation of Clinical Chemistry (IFCC) and the Clinical and International Standards Institute (CISI) recommend establishing reference intervals (RIs) for each specific area^14–16^. Although the concept of RI described in 1969^17,18^. In addition, several studies established RIs in Middle Eastern countries^11,19,20^. Nevertheless, the RIs ​​in Yemen depend on scientific textbooks or manufactured kits, which rely on studies conducted in other countries populations. This situation underscores the need for establishing specific RIs for the Yemeni population. Thus, this study aimed to establish special LFT-RIs for adults in Ibb City -Yemen.

## Subjects and methods

### Study population and participants

The participants were randomly selected and recruited from well-adults, aged 18 to 70. The study sample size was determined according to the Clinical and Laboratory Standards Institute (CLSI) policy, which guides at least 120 healthy individuals to establish RIs with 95% confidence intervals (CI) per group^21–23^. To maximize the trust, the researchers raised the participant’s number to 523.

From 523 healthy volunteers were included, 390 were ultimately selected for enrollment, while 133 (25.43%) were excluded.

The exclusion criteria included individuals with a history of alcohol consumption, those taking medications or supplements, and individuals with chronic diseases (liver diseases, hypertension, renal diseases, heart disease, arthritis, thyroid disorders, and diabetes). Other reasons for exclusion were inflammatory disorders (like tonsillitis, appendicitis, and fever), allergic disease, skin rash, donation or receipt blood within the previous year, and a positive result from serological analyses (CRP, HBV, and HCV).

The female volunteers were excluded if they were pregnant, menstruating, breastfeeding, or within one year postpartum. As a result, 390 healthy volunteers, 211 males and 179 females, participated.

### Sampling methods and analysis

The participants were randomly selected and were administered a questionnaire that gathered information on age, weight, gender, residence, and exclusion criteria.

After an overnight fast, 5 ml of venous blood samples were assembled under aseptic states. The blood samples were then shipped in a dry ice box to the laboratory within one hour. Upon arrival, the samples were centrifuged for 5 min at 3000 rpm to separate the sera.

The serum was analyzed to determine RIs of commonly used LFT, alanine aminotransferase (ALT), aspartate aminotransferase (AST), alkaline phosphatase (ALP), total protein (TP), albumin (ALB), total bilirubin (TBIL) and direct bilirubin (DBIL) using Mindray BS-240 Automatic Analyzer (Shenzhen Mindray Bio-Medical Electronics Co., China). Moreover, CRP was detected by utilizing the Spinreact CRP-latex reagent (Spinreact, Girona Spain). Additionally, HBV and HCV were detected by using a Healgen rapid test cassette (Healen, Houston, USA).

All analyses were conducted were analyzed according to manufacturer’s guidelines at the Biochemistry department, Alfa Medical Laboratories, Ibb City, spanning from March 2023 to December 2023 (A cross-sectional study).

### Ethical considerations

The ethical clearance for this research was granted by the Medical Laboratories Department, Faculty of Medicine and Health Sciences, Ibb University ethical committee after due process was followed (Reference No.: MDL-MHS-IBBU/IBA001/2023 on February 6. 2023). The Ethics Research Committee was aligned with the Declaration of Helsinki for the Protection of Human Subjects. The autonomy of participants was respected, and they were informed of the study’s risks, benefits, and objectives. Informed consent was obtained from all subjects.

### Data analysis

The data gathered from the screening tests and questionnaires were computed by GraphPad Prism 8.0.1 (GraphPad Inc. USA). The median, mean, and nonparametric 95% confidence intervals (CI) of RIs (2.5th and 97.5th percentiles) were computed for the common LFT following the guidelines set by CLSI^24^. Outlier values were detected by the ROUT (Q = 1%) method. The data normality was determined using Kolmogorov-Smirnov test (KST), Shapiro-Wilk test (SWT), and Anderson-Darling test (ADT). An ANOVA and Kruskal Wallis test (KWT) were used to test an association’s significance. *P* value ≤ 0.05 was estimated as statistically significant.

## Results

### Demographic characteristics of participants

The demographic characteristics of contributors were revealed in Table 1. 390 healthy adults (211 males and 179 females) aged 18 to 70 years with a mean of 29.09 ± 10.15 years were included for establishing RIs of commonly used LFT parameters. The mean body weight was 57.71 ± 10.14 Kg, and 85.38% of participants were urban residents.

**Table 1:** Demographic characteristics of participants.

Characteristics (No.=390)	Mean± (SD)
Age	29.09 ± 10.15 years
Weight	57.71 ± 10.14 Kg
Gender	No. (%)
Male	211 (54.10%)
Female	179 (45.90)
Residence	No. (%)
Urban	333 (85.38%)
Rural	57 (14.62%)

## Liver function tests RIs based on gender of participants

The mean, median, and RIs (2.5th and 97.5th percentiles) are shown in Table 2. The median and RIs of common LFT for males and females were DBIL (mg/dl) 0.2900 (0.0620–0.6480 mg/dl) and 0.1850 (0.000–0.4925 mg/dl), TBIL (mg/dl) 0.5000 (0.1880–1.116 mg/dl) and 0.3600 (0.1918–0.7300 mg/dl), ALB (g/dl) 4.200 (3.800–4.970 g/dl) and 4.100 (3.700–4.555 g/dl), total protein (g/dl) 7.400 (6.600–8.300 g/dl) and 7.350 (6.448–8.253 g/dl), ALP (U/L) 125.0 (76.35–199.0 U/L), and 98.00 (58.30–156.5 U/L), AST 28.00 (10.00–53.88 U/L) and 28.00 (11.00–45.20 U/L), and ALT (U/L) 19.50 (10.00-39.23 U/L) and 14.00 (7.000–29.80 U/L), respectively. RIs for males and females of the DBIL, TBIL, ALB, ALP, and ALT were statistically significant.

**Table 2:** Mean, median and 95% RIs of common liver function tests among healthy adults based on gender.

Parameters	Gender	Mean	Median	95% CI	RIs	*P*-value
DBIL (mg/dl)	Combined	0.2578	0.2500	0.2400–0.2700	0.000–0.6438	< 0.0001*
	Male	0.3170	0.2900	0.2700–0.3200	0.0620–0.6480	
	Female	0.1783	0.1850	0.1500–0.2100	0.000–0.4925	
TBIL (mg/dl)	Combined	0.4738	0.4400	0.4200–0.4500	0.1940–0.9560	< 0.0001*
	Male	0.5386	0.5000	0.4600–0.5400	0.1880–1.116	
	Female	0.3940	0.3600	0.3400–0.4000	0.1918–0.7300	
ALB (g/dl)	Combined	4.231	4.200	4.100–4.200	3.778–4.900	< 0.0001*
	Male	4.328	4.200	4.200–4.300	3.800–4.970	
	Female	4.105	4.100	4.100–4.150	3.700–4.555	
TP (g/dl)	Combined	7.384	7.400	7.300–7.400	6.500–8.300	0.7238
	Male	7.401	7.400	7.300–7.400	6.600–8.300	
	Female	7.363	7.350	7.200–7.500	6.448–8.253	
ALP (U/L)	Combined	116.6	113.0	109.0–117.0	64.58–198.4	< 0.0001*
	Male	127.9	125.0	121.0–130.0	76.35–199.0	
	Female	100.3	98.00	92.00–100.0	58.30–156.5	
AST (U/L)	Combined	27.73	28.00	27.00–29.00	10.00–48.00	0.5373
	Male	27.35	28.00	25.00–30.00	10.00–53.88	
	Female	28.50	28.00	27.00–29.00	11.00–45.20	
ALT (U/L)	Combined	17.98	16.50	16.00–18.00	8.000–35.03	< 0.0001*
	Male	20.40	19.50	18.00–20.00	10.00–39.23	
	Female	15.24	14.00	13.00–15.00	7.000–29.80	

## Liver function tests RIs based on residence of participant

Regarding residence differences in RIs of the LFT, the RIs of TBIL and TP in the urban group are higher than in rural residents. The RIs of DBIL, ALB, ALP, AST, and ALT in the rural group are higher than in urban residents as presented in Table 3. Furthermore, the DBIL, ALB, ALP, and ALT parameters were statistically significant.

**Table 3:** Mean, median and 95% RIs of common liver function tests among healthy adults based on residence.

Parameters	Residence	Mean	Median	95% CI	RIs	*P*-value
DBIL (mg/dl)	Combined	0.2578	0.2500	0.2400–0.2700	0.000–0.6438	0.0291*
	Urban	0.2503	0.2400	0.2300–0.2600	0.000–0.6478	
	Rural	0.3020	0.2800	0.2600–0.3400	0.000–0.6718	
TBIL (mg/dl)	Combined	0.4738	0.4400	0.4200–0.4500	0.1940–0.9560	0.2293
	Urban	0.4660	0.4300	0.4100–0.4400	0.2000–0.9500	
	Rural	0.4989	0.4700	0.4200–0.5600	0.1670–0.9490	
ALB (g/dl)	Combined	4.231	4.200	4.100–4.200	3.778–4.900	0.0020*
	Urban	4.198	4.100	4.100–4.200	3.728–4.900	
	Rural	4.374	4.300	4.200–4.600	3.745–5.010	
TP (g/dl)	Combined	7.384	7.400	7.300–7.400	6.500–8.300	0.9994
	Urban	7.386	7.400	7.300–7.400	6.500–8.300	
	Rural	7.374	7.400	7.200–7.500	6.500–8.155	
ALP (U/L)	Combined	116.6	113.0	109.0–117.0	64.58–198.4	< 0.0001*
	Urban	113.3	110.0	105.0–113.0	63.35–193.6	
	Rural	136.6	131.0	122.0–143.0	73.00–212.4	
AST (U/L)	Combined	27.73	28.00	27.00–29.00	10.58–48.00	0.5357
	Urban	28.00	28.00	27.00–29.00	10.15–48.00	
	Rural	26.16	27.00	19.00–31.00	9.900–52.50	
ALT (U/L)	Combined	17.98	16.00	16.00–18.00	8.000–35.03	0.0013*
	Urban	17.23	16.00	15.00–18.00	8.000–31.40	
	Rural	21.47	20.00	16.00–22.00	10.35–40.95	

## Discussion

**Table 4:** Comparison of RIs obtained in the current study with studies in different countries.

Parameters	Gender	Reference interval (RIs)									
		Current study	Saudi Arabia^25^	Egypt^19^	Turkey^20^	India^18^	South Africa^26^	Ethiopia^27^	Cameroon^28^	Tanzania^29^	Ghana^30^
DBIL (mg/dl)	C	0.00–0.6438	NA	NA	0.9 − 0.51	NA	NA	NA	0.1–1.2	0.042–0.48	0.046–0.234
	M	0.062–0.6480	NA	0.04–0.37	NA	NA	NA	NA	NA	0.054–0.49	0.052–0.239
	F	0.00–0.4925	NA	0.03–0.21	NA	NA	NA	NA	NA	0.41 − 0.34	0.041–0.222
TBIL (mg/dl)	C	0.1940–0.9560	NA	NA	0.23–1.32	NA	0.22–1.29	0.1–1.23	0.4–3.3	0.30–2.397	0.17–1.51
	M	0.1880–1.116	0.21–1.30	NA	0.15–1.20	0.36–1.38	NA	0.1–1.3	NA	0.35–2.456	0.22–1.87
	F	0.1918–0.7300	0.13–0.90	NA	0.3–0.72	0.23–1.012	NA	0.1–1.2	NA	0.26–1.83	0.16–1.56
ALB (g/dl)	C	3.778–4.900	3.9–5	3.6–5.1	3.46–4.9	NA	NA	NA	NA	3.56–5.04	3.30–4.99
	M	3.800–4.970	NA	NA	NA	NA	3.7–4.9	NA	NA	3.70–5.07	3.27–4.98
	F	3.700–4.555	NA	NA	NA	3.6–4.7	3.4–4.7	NA	NA	3.557–4.927	3.35–5.04
TP (g/dl)	C	6.500–8.300	6.2–7.7	6.0–8.2	6.0–8.1	6.8–8.6	6.3–8.2	5.4–8.9	NA	6.63–8.51	5.06–8.67
	M	6.600–8.300	NA	NA	NA	NA	NA	5.5–8.1	NA	6.72–8.52	4.67–8.64
	F	6.448–8.253	NA	NA	NA	NA	NA	5.2–9.0	NA	6.58–8.55	5.52–8.69
ALP (U/L)	C	64.58–198.4	39–114	NA	35–107	NA	NA	35–342	52.6–251.1	45.6–158.4	85–241
	M	76.35–199.0	NA	45–131	NA	41–111	47–126	55–343	NA	45.4–170.4	101–353
	F	58.30–156.5	NA	38–126	NA	NA	41–122.5	50–326	NA	45.3–155.0	82–293
AST (U/L)	C	10.00–48.00	NA	NA	NA	NA	NA	16–60	8.7–40.7	14.3–48.1	14–51
	M	10.00–53.88	11–28	12–38	9–38	20–53	14–42	19–63	NA	15.2–53.4	17–60
	F	11.00–45.20	10–24	10–32	9–27	17–39	12–31	16–59	NA	13.5–35.2	13–48
ALT (U/L)	C	8.000–35.03	NA	NA	NA	NA	NA	11–48	5.2–29.1	7.7–48.3	7–51
	M	10.00–39.23	7–39	4–66	5–53	15–74	3.8–40.8	14–49	NA	9.1–55.3	8–54
	F	7.000–29.80	5–18	3–43	4–32	10–37	2.8–22.3	7.9–48	NA	6.7–44.9	6–51

*C* Combined, *M* Male, *F* Female, *DBIL* direct bilirubin, *TBIL* total bilirubin, *ALB* albumin, *TP* total protein, *AST* aspartate aminotransferase, *ALT* alanine aminotransferase, *RIs* reference intervals, *mmol/L* millimole per liter, *g/dl* gram per deciliter, *U/L* units per liter, *NA* not available.

As shown in Table 4, based on gender, this study revealed that the male RIs for DBIL, TBIL, ALB, and TP were higher than RIs in females. This finding for DBIL agreed with studies conducted in Egypt^19^, Tanzania^29^, and Ghana^30^. Likewise, the RI of TBIL was higher in males, aligning with studies from Saudi Arabia^11^, India^18^, Tanzania^29^, and Ghana^30^. Moreover, we noticed that the RI of ALB in males was greater than females, which is harmonic with previous evidence in South Africa and Tanzania^26,29^, while a study in Ghana reported that the RI of ALB was higher in females than in males^30^. Furthermore, our findings regarding the RI for TP disagreed with a study conducted in Ghana^30^. The variation in RIs can be influenced by lifestyle factors, environmental conditions, analytical methods^31^, and demographic characteristics such as age, sex, body weight, dietary habits, and hormone levels^31–33^.

In the current study, the upper RI of DBIL was lower than the value in Cameroon^28^ and higher than those reported from Turkey^20^, Ghana^30^, and Tanzania^29^. Regarding TBIL, the RI was lower than the RIs in South Africa, Ethiopia, Cameroon, Tanzania, Turkey, and Ghana^20,26–30^. The RI of ALB in the study was almost compatible with reports from Saudi Arabia, Egypt, and Tanzania^19,25,29^, as well as almost compatible with the upper limit of reports from Turkey and Ghana^20,30^. Furthermore, the RI of TP was almost higher than RIs from Saudi Arabia, Egypt, Turkey, and South Africa^11,19,20,26^ but was lower than RI of TP in India and Tanzania^18,29^. These variations suggest that the RIs established in other countries may not applicable to Yemeni population.

In the present study, the RIs of liver enzymes like ALP, AST, and ALT were also examined. The observed RI for ALP was higher than those reported in Saudi Arabia, Turkey, and Tanzania^11,20,29^, but lower than RI documented in Ghana^30^. Moreover, the upper limit of ALP in Ethiopia and Cameroon was higher than our result^27,28^. Furthermore, we found the ALP in males was higher than the value in females; which is harmonic with results from other studies in Egypt^19^, South Africa^26^, Ethiopia^27^, Tanzania^29^, and Ghana^30^.

For AST, the RI was obtained and noted to be higher than the RI reported from Cameroon^28^; but lower than RI from Ghana and Ethiopia^27,30^. Additionally, we found the RI of AST in males was higher than RI in females, which is harmonic with results from various studies conducted in Saudi Arabia^25^, Egypt^19^, India^18^, South Africa^26^, Ethiopia^27^, Tanzania^29^, and Ghana^30^.

Regarding ALT, our results showed that the RI of ALT was higher than the RI in Cameroon^28^; but lower than those from Ethiopia^27^. The RI of ALT was found to be higher in males than females. These findings were similar to RIs in the studies in Saudi Arabia^11^, Egypt^19^, Turkey^20^, India^18^, South Africa^26^, Ethiopia^27^, Tanzania^29^, and Ghana^30^. Additionally, we noticed that the upper reference limits of ALT in males and females were lower than the upper reference limit of ALT reported in Tanzania^29^ and Ghana^30^. The variation of RIs for DBIL, TBIL, ALB, TP, ALP, AST and ALTin different studies may be related to the demographic characteristics of the participants including sex, age, body weight, dietary pattern, and sex hormone^32–34^. Also, the variations in liver function tests are affected by lifestyle, environmental factors, and analytical methods^31^.

Regarding residence difference in RIs, RIs of DBIL, ALB, ALP, AST, and ALT were higher in rural than in urban. Statistically significant differences were observed for DBIL, ALB, ALP, and ALT. These findings might be influenced by the small number of the rural residence group (14.62%). These results merit further study to establish the RIs of LFT.

In conclusion, this research established the RIs for LFTs, highlighting significant gender differences, where the results displayed significant in DBIL, TBIL, ALB, ALP, and ALT between males and females. This study is pioneering the establishment of RIs for LFTs in Yemen, particularly in Ibb City. It emphasized the differences in RIs between the Yemeni population and those of other countries, which should be considered when interpreting laboratory diagnostic results.

To establish RIs for LFTs in the Yemeni population, it is necessary to conduct a multicenter study with a larger sample size. Further research is recommended to address the limitations, particularly relating to the RIs for the LFT in children and neonates, as well as the impact of body mass index (BMI) on RIs of LFTs.

## Data Availability

The data sets generated during and/or analyzed during the current study are available from the corresponding author upon reasonable request.
